# Identifying the Prognostic Risk Factors of Synaptojanin 2 and Its Underlying Perturbations Pathways in Hepatocellular Carcinoma

**DOI:** 10.1080/21655979.2021.1890399

**Published:** 2021-03-01

**Authors:** Rui Zhang, Wei-Jia Mo, Lan-Shan Huang, Ji-Tian Chen, Wei-Zi Wu, Wei-Ying He, Zhen-Bo Feng

**Affiliations:** a Department of Pathology, First Affiliated Hospital of Guangxi Medical University, Nanning, Guangxi Zhuang Autonomous Region, China; b Department of Pathology, People’s Hospital of Ling Shan, Ling Shan, Guangxi Zhuang Autonomous Region, China

**Keywords:** Synaptojanin 2, hepatocellular carcinoma, nomogram predicting, differentially coexpressed genes, metabolism

## Abstract

Synaptojanin 2 (SYNJ2) regulates cell proliferation and apoptosis via dephosphorylating plasma membrane phosphoinositides. Aim of this study is to first seek the full-scale expression levels and potential emerging roles of SYNJ2 in hepatocellular carcinoma (HCC). We systematically analyzed SYNJ2 mRNA expression and protein levels in HCC tissues based on large-scale data and in-house immunohistochemistry (IHC). The clinical significance and risk factors for SYNJ2-related HCC cases were identified. A nomogram of prognosis was created and its performance was validated by concordance index (C-index) and shown in calibration plots. Based on the identified differentially coexpressed genes (DCGs) of SYNJ2, enriched annotations and potential pathways were predicted, and the protein interacting networks were mapped. Upregulated SYNJ2 in 3,728 HCC and 3,203 non-HCC tissues were verified and in-house IHC showed higher protein levels of SYNJ2 in HCC tissues. Pathologic T stage was identified as a risk factor. Upregulated mRNA levels and mutated SYNJ2 might cause a poorer outcome. The C-index of the nomogram model constructed by SYNJ2 level, age, gender, TNM classification, grade, and stage was evaluated as 0.643 (95%CI = 0.619–0.668) with well-calibrated plots. A total of 2,533 DCGs were extracted and mainly functioned together with SYNJ2 in metabolic pathways. Possible transcriptional axis of CTCF/POLR2A-SYNJ2/INPP5B (transcription factor-target) in metabolic pathways was discovered based on ChIP-seq datasets. In summary, transcriptional regulatory axis CTCF/POLR2A-SYNJ2 might influence SYNJ2 expression levels. Increased SYNJ2 expression level could be utilized for predicting HCC prognosis and potentially accelerates the occurrence and development of HCC via metabolic perturbations pathways.

## Graphical abstract

**Figure:**
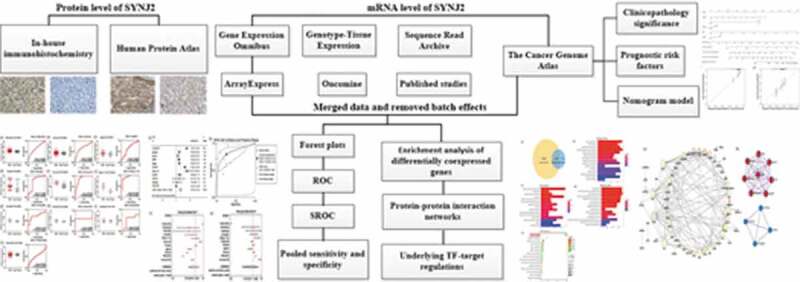

## Introduction

Hepatocellular carcinoma (HCC) is a malignant tumor prevalent worldwide and is the fourth leading cause of cancer-related deaths. There are approximately 841,000 new cancer cases and 782,000 cancer-related deaths each year [1]. Treatments such as surgical resection, liver transplant, transcatheter arterial therapy, and systemic chemotherapy are mainly adopted for HCC patients [2,3]. Among these, surgery is the most effective option [4]. However, many HCC patients are diagnosed at an advanced stage and have a poor prognosis due to rapid progression and insidious early symptoms [5]. The treatment effect is so limited for patients with advanced HCC that over 50% of patients will die within one year [6,7]. Therefore, it is of great significance to find the possible biomarkers and regulatory mechanisms of HCC for better prognosis and effective therapeutic targets.

Synaptojanin 2 (SYNJ2) is a gene located on chromosome 6q25.3 encoding a phosphoinositide phosphatase and acts primarily via protein–protein interaction. SYNJ2 has three domain structures that play roles in regulating ionic channels and transporters, cell proliferation and apoptosis, cytoskeleton rearrangement, and membrane vesicle trafficking by dephosphorylating plasma membrane phosphoinositides [8]. SYNJ2 is necessary for the regulation of clathrin-mediated endocytosis, particularly at an early stage, which can promote the formation of astrocytic lamellipodia [9–11]. In various types of tumor cells, SYNJ2 has been found to play an essential role in invasiveness capability [12]. The clathrin-mediated endocytosis and formation of invadopodia mediated by SYNJ2 are considered to be closely related to the invasive behavior of tumor cells. Highly enriched SYNJ2 in invadopodia is likely to regulate key proteins (such as cortactin and TKS5) by the phosphatidylinositol metabolism process to affect the invasion of malignant cells [11,13–15]. Previous studies have revealed that abnormal expression of SYNJ2 plays different roles in several cancers. In colorectal cancer, an increased protein level of SYNJ2 was reported, and the SYNJ2 variant rs9365723 was taken to be a risk factor in Chinese patients. SYNJ2 probably plays a key role in the tumorigenesis of colorectal cancer [16,17]. In breast cancer, overexpression or amplification of SYNJ2 was considered to be closely correlated with shorter survival times. Additionally, formation of cellular lamellipodia and invadopodia promoted by SYNJ2 might affect the migration and invasion of cancer cells [18]. Dysregulated SYNJ2 levels potentially facilitate development and deteriorate the prognosis of malignancies for patients. However, the influence of mRNA and protein levels on prognosis and the underlying regulatory mechanisms of SYNJ2 in HCC still remain unknown. This study will explore the clinical value and potential key molecular mechanisms of SYNJ2 in HCC.

Here, we have used clinical specimens of and abundant public data on HCC tissues to explore the clinical and prognostic significances of SYNJ2 in HCC. The risk factors were analyzed, and a nomogram model was constructed based on a multivariate Cox regression analysis. Both the enrichment pathways and the transcriptional regulatory axis in which SYNJ2 involved were obtained.

## Materials and methods

### Clinical value of SYNJ2 in HCC samples

The RNA sequencing data from 371 HCC and 50 para-carcinoma liver tissues were downloaded from The Cancer Genome Atlas (TCGA) database along with the corresponding clinical parameters. The level 3 mRNA expression values of SYNJ2 were extracted. We graphed the corresponding receiver operating characteristic (ROC) curves, and the areas under the curves (AUCs) were calculated. The prognostic capability of SYNJ2 was assessed by Kaplan-Meier survival estimation.

### Risk factors and nomogram predicting

Cox regression models were constructed to estimate the potential risk factors based on SYNJ2 levels and the clinical parameters of 371 HCC patients from the TCGA database. Clinical characteristics with different SYNJ2 levels were selected and incorporated into prognostic nomogram predicting models. The predictive performance of each nomogram was validated via calibration. The analyses were performed using different R packages, including ‘survival’ and ‘rms’.

### Expression of SYNJ2 in public databases

The SYNJ2 levels in various tissues and related cell lines were separately obtained from the Gene Expression Profiling Interactive Analysis (GEPIA, http://gepia.cancer-pku.cn) and Cancer Cell Line Encyclopedia (CCLE, https://portals.broadinstitute.org/ccle) databases. SYNJ2 expression data from normal human liver tissues were downloaded from the Genotype-Tissue Expression (GTEx, https://gtexportal.org) project. We further comprehensively collected SYNJ2 expression data from the Sequence Read Archive (SRA, https://www.ncbi.nlm.nih.gov/sra), Gene Expression Omnibus (GEO, https://www.ncbi.nlm.nih.gov/geo), ArrayExpress (https://www.ebi.ac.uk/arrayexpress/), and Oncomine (https://www.oncomine.org/) databases as well as from published studies related to the levels of SYNJ2 in HCC using the following search strategy: (hepatocellular OR HCC OR hepatic OR liver) AND (tumor OR tumor OR carcinoma OR cancer OR neoplas* OR malignan*).

## Data extraction and processing

Datasets and studies meeting these three criteria were included: 1) the species studied was human; 2) each dataset contained HCC and nontumorous liver tissues; and 3) the expression value of SYNJ2 could be extracted. SYNJ2 expression data were then collected and normalized by log2 transformation. The batch effects among different platforms were removed using removeBatchEffect function from the R package ‘Limma’, and the differentially expressed genes (DEGs) of HCC were also analyzed using the ‘Limma’ package (|logFC| > 1, adjusted P-value < 0.05). The SYNJ2 expressions from TCGA and GTEx were combined to create a group that contained more noncancerous clinical samples. The batch effects between these two datasets were eliminated using the aforementioned function, and the DEGs of the combined group were calculated via the R package ‘Limma Voom’. Scatter plots and ROC curves of the SYNJ2 expression conditions were generated.

## Identifying SYNJ2 clinical potential based on combined data

We comprehensively assessed the discriminatory and diagnostic abilities of SYNJ2 for HCC by combining all data. Stata 12.0 (StataCorp LLC, College Station, TX, USA) was used to calculate the standardized mean difference (SMD) with a 95% confidential interval (CI). The heterogeneity was determined by a chi-square test and I^2^ statistical values, and a fixed-effects model was chosen if the P-value was greater than 0.01 or the I^2^ values were less than 50%; otherwise, a random-effects model was used. The pooled sensitivity and specificity were all calculated and visualized with forest plots. A summary ROC (SROC) plot was created, and an AUC > 0.7 indicated reliable discrimination.

## Genetic alterations of SYNJ2

Based on the cBioPortal database, we analyzed the genetic alterations and mutation types of SYNJ2 in HCC. Survival curves were constructed to visually demonstrate the relationships between genetic alterations and the prognoses of HCC patients.

## Differentially coexpressed genes of SYNJ2 in HCC

To further discover the potential molecular mechanisms, we screened the coexpressed genes of SYNJ2 from the included HCC datasets. Pearson’s correlation analysis of SYNJ2 and other genes in different platforms were respectively calculated by using cor.test function of R software. The genes with a Pearson correlation coefficient score |r| ≥ 0.8 and P-value < 0.05 were selected as coexpressed genes of SYNJ2 in HCC. In addition, the differentially coexpressed genes (DCGs) were selected from the intersections of coexpressed genes and DEGs.

## Functional characterization of differentially coexpressed genes

To identify potential mechanisms of HCC, we attempted to identify the enriched gene ontology (GO) terms and Kyoto Encyclopedia of Genes and Genomes (KEGG) signaling pathways of SYNJ2-relevant DCGs via the R package ‘clusterProfiler’. The protein–protein interaction (PPI) networks were constructed with STRING (https://string-db.org/), and we used Cytoscape 3.7.2 to identify the highly interconnected proteins based on Molecular Complex Detection (MCODE) clustering algorithm.

## Potential transcriptional regulations

The transcriptional associations of interactions between SYNJ2 and DCGs were discovered via the Database of Human Transcription Factor Targets (hTFtarget, http://bioinfo.life.hust.edu.cn/hTFtarget), which contained 7,190 experiment samples of 659 human transcription factors (TFs) based on ChIP-seq data. Meanwhile, the specific diseases related to TF regulation were found from the KEGG Orthology Based Annotation System (KOBAS, http://kobas.cbi.pku.edu.cn/kobas3).

## In-house immunohistochemistry and public SYNJ2 protein expression

We collected 285 HCC and 37 nontumorous formalin-fixed paraffin-embedded (FFPE) tissues from the Pathology Department of the First Affiliated Hospital of Guangxi Medical University between 1 January 2014, and 31 December 2015. The FFPE tissues were sliced into 4 µm sections (RM2235, Leica, Germany), and a rabbit polyclonal antibody against synaptojanin-2 (orb513930, Biorbyt, Cambridge, UK) was chosen as the primary antibody. The immunohistochemistry (IHC) procedure was performed as previously reported [19]. Two aspects could have influenced the total score of IHC staining (Q): 1) staining degree (S): 0 (no staining), 1 (weak staining), 2 (moderate staining), and 3 (strong staining); and 2) percentage of positive cells (P): 0 (<5%), 1 (5–25%), 2 (26–50%), 3 (51–75%), and 4 (>75%). Both S and P were calculated under objective magnification 10X. Q was calculated as S × P, with a maximum score of 12, and a moderate score of 6 was used to separate the positive (≥6) and negative samples (<6). Three pathologists assessed the staining results independently. The current study was approved by the Ethics Committee of the First Affiliated Hospital of Guangxi Medical University. The details of this study were provided, and informed consent was obtained from all patients or their family members. Furthermore, the protein expression data of SYNJ2 in HCC were obtained from the Human Protein Atlas (HPA).

## Statistical analysis

The data in the current study were statistically analyzed and graphed using SPSS 22.0 (IBM, New York, USA), GraphPad Prism 7.0 (GraphPad Software, San Diego, CA, USA), and Stata 12.0 (StataCorp LLC, College Station, TX, USA). Comparisons between groups were estimated by a Student’s t-test. Pearson’s correlation coefficients were calculated to examine the relationships of SYNJ2 and its coexpressed genes. Values of P less than 0.05 were considered statistically significant.

## Results

In this study, the mRNA level of SYNJ2 was investigated based on large-scale public data of HCC tissues and the protein level was validated using in-house IHC. The clinicopathology characteristics and risk factors were analyzed via TCGA data. A nomogram model was built to explore the ability to predict the prognosis of dysregulated SYNJ2 in HCC patients. The underlying mechanism influenced by SYNJ2 along with its differentially coexpressed genes was uncovered utilizing enrichment analysis. For deeper insight into the regulatory mechanism, we studied the regulatory axis of transcription factors and SYNJ2 in HCC based on experimentally verified ChIP-seq data.

## SYNJ2 levels in tissues and cell lines

**Figure 1.**
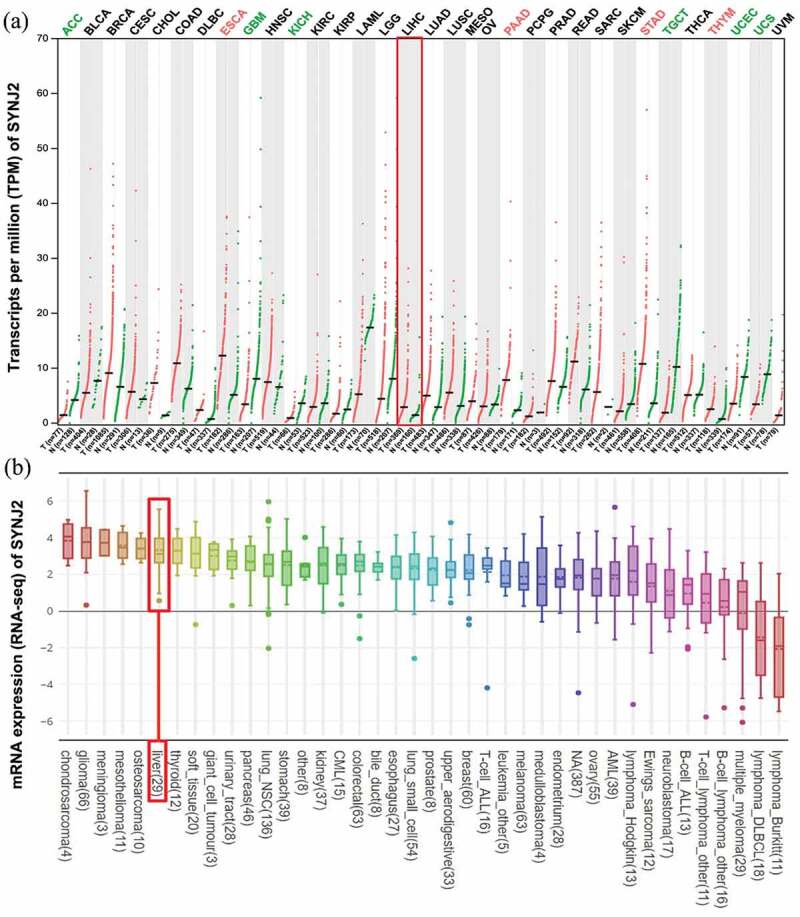
**SYNJ2 levels in different tissues and cell lines**. (a) SYNJ2 levels in various cancer and non-cancerous tissues. The expression of liver HCC and non-HCC tissues were marked in the red box. The red dots and green dots, respectively, represent the expression of HCC and non-HCC samples. (b) Expression of SYNJ2 in liver-related tumorous cell lines. The box plot was marked in the red box

To investigate the mRNA expression of SYNJ2 in various malignancies and cell lines, we obtained data from the GEPIA and CCLE databases. We found that compared to that in noncancerous tissues, SYNJ2 expression in 15 types of malignancies was decreased, while in 16 tumors, including liver HCC, SYNJ2 expression was increased (Figure 1(a)). Similarly, the mRNA expression levels of SYNJ2 in liver cancer cell lines were also higher than those in the other cell lines (Figure 1(b)).

## Clinicopathological significance of SYNJ2 in HCC

**Figure 2.**
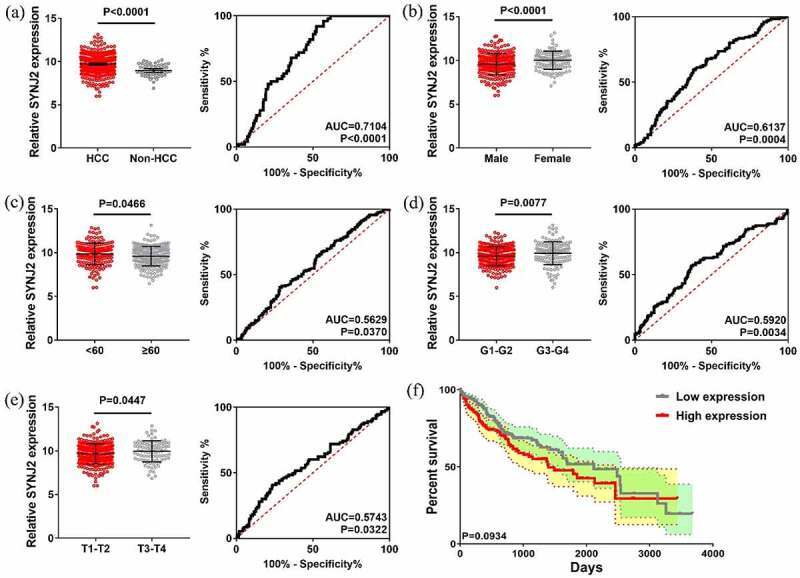
**Levels and efficacy of SYNJ2 in clinical parameters**. (a) Scatter plots and ROC curve of SYNJ2 in HCC and non-HCC tissues. (b) Scatter plots and ROC curve of SYNJ2 in male and female HCC patients. (c) Scatter plots and ROC curve of SYNJ2 in young (<60) and elder (≥60) HCC cases. (d) Scatter plots and ROC curve of SYNJ2 in G1-G2 and G3-G4 HCCs. (e) Scatter plots and ROC curve of SYNJ2 in T1-T2 and T3-T4 HCCs. (f) Overall survival status of HCCs with different SYNJ2 levels

We analyzed the clinical and pathological data of SYNJ2 in 371 HCC and 50 non-HCC cases. SYNJ2 was found to be expressed higher in HCC tissues than in normal tissues and significantly correlated with sex, age, grade, and pathologic T stage with a P-value <0.05 (Table 1). The association of SYNJ2 levels with different significant clinical variables was determined with ROC curves, and the levels of SYNJ2 are displayed (Figure 2(a-e)). SYNJ2 was upregulated in HCC tissues (P < 0.0001), female patients (P < 0.0001), elderly patients (P = 0.0466), G3-G4 HCCs (P = 0.0077), and T3-T4 HCCs (P = 0.0447). SYNJ2 levels in HCC and non-HCC tissues showed the highest AUC value of 0.7104 (P < 0.0001) which means that SYNJ2 may has high value for HCC early diagnosis. As can be seen in Figure 2(f), there was a clear trend of better overall survival outcomes with lower SYNJ2 expression in HCCs.

**Table 1.** Clinical and pathological parameters of SYNJ2 in HCCs

Variables	Terms	N	Mean ± SD	t	P-value
Tissue	HCC	371	9.7224 ± 1.1839	6.2447	1.368E-08
	Non-HCC	50	8.9827 ± 0.7161		
Gender	Male	250	9.5726 ± 1.2235	−3.5583	4.220E-04
	Female	121	10.0320 ± 1.0356		
Age	≥60	201	9.6082 ± 1.1112	1.9967	0.0466
	<60	169	9.8541 ± 1.2574		
Grade	G1-G2	232	9.6053 ± 1.0898	−2.6794	0.0077
	G3-G4	134	9.9472 ± 1.3124		
Stage	I–II	257	9.6183 ± 1.1747	−1.9216	0.0554
	III–IV	90	9.8987 ± 1.1884		
Pathologic T	T1-T2	275	9.6574 ± 1.1664	−2.0143	0.0447
	T3-T4	93	9.9421 ± 1.2139		
Pathologic N	N0	252	9.7033 ± 1.1966	−1.2379	0.2169
	N1	4	10.4466 ± 0.6153		
Pathologic M	M0	266	9.6866 ± 1.1865	0.3093	0.7573
	M1	4	9.5014 ± 1.3792		

HCC: hepatocellular carcinoma. SD: standard deviation.

## Predicted risk factors and nomogram development

**Figure 3.**
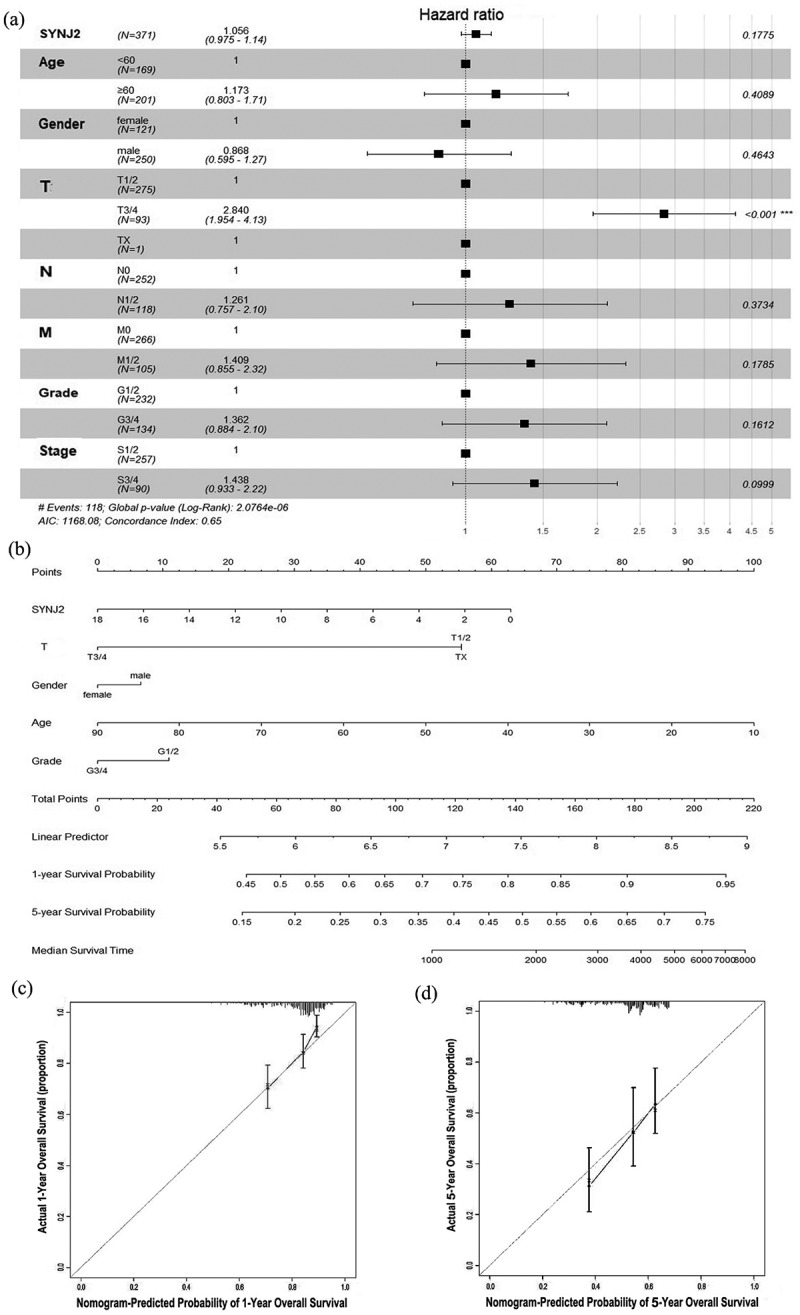
**Visualization of multivariate Cox regression analyses and prognostic nomogram predicting**. (a) Forest plot of hazard ratio showing the estimation of SYNJ2 expression and various clinicopathological parameters in HCC. (b) Nomogram predicting overall survival for significant clinicopathological parameters of HCC patients, including SYNJ2 level, gender, age, grade and T stage. (c, d) Calibration plots of the nomogram predictive performance of 1- and 5-year overall survival based on five variables (SYNJ2 level, gender, age, grade and T stage). The y-axis and x-axis separately represent actual and nomogram-predicted overall survival rate. The 45° gray line represents an ideal prediction and the black line means the nomogram-predicted performance

We investigated the association between HCC and different clinical variables, including SYNJ2 level, age (<60 and ≥60), gender (male and female), T (TX, T1/2, and T3/4), N (N0 and N1/2), M (M0 and M1/2), grade (G1/2 and G3/4), and stage (S1/2 and S3/4). The forest plots of hazard ratio (HR) indicated that the T stage is a risk factor in HCC cases. The T3/4 stage was considered to be a significant increased risk in HCC (HR = 2.8307, confidence interval (CI) 95% = 1.9467–4.1160, P < 0.001) (Figure 3(a)). We could also infer that low SYNJ2 in HCC patients potentially suggests a smaller risk. Furthermore, the nomogram prediction model was constructed based on five significant clinicopathological variables of SYNJ2 in HCC, including SYNJ2 expression level, T (TX, T1/2, and T3/4), gender (male and female), age (<60 and ≥60), and grade (G1/2 and G3/4). The total points need to be summed up from each variable by drawing a vertical line upwards from the point axis. The 1-year and 5-year overall survival probability and median survival time could be estimated by drawing a vertical line downwards from the total point axis (Figure 3(b)). Although SYNJ2 showed no significant results in the multivariate Cox regression analysis, in prognostic nomogram model, lower SYNJ2 could accumulate higher survival scores which means that SYNJ2 may indirectly influence and potentially improve the prognosis of HCC patients. The calibration diagram showed a satisfactory consistency between the nomogram-predicted value and actual value in 1-year and 5-year overall survival (OS) probability, the C-index of the nomogram was 0.643 (95% CI = 0.619–0.668), as shown in Figure 3(c).

## SYNJ2 expression datasets

**Figure 4.**
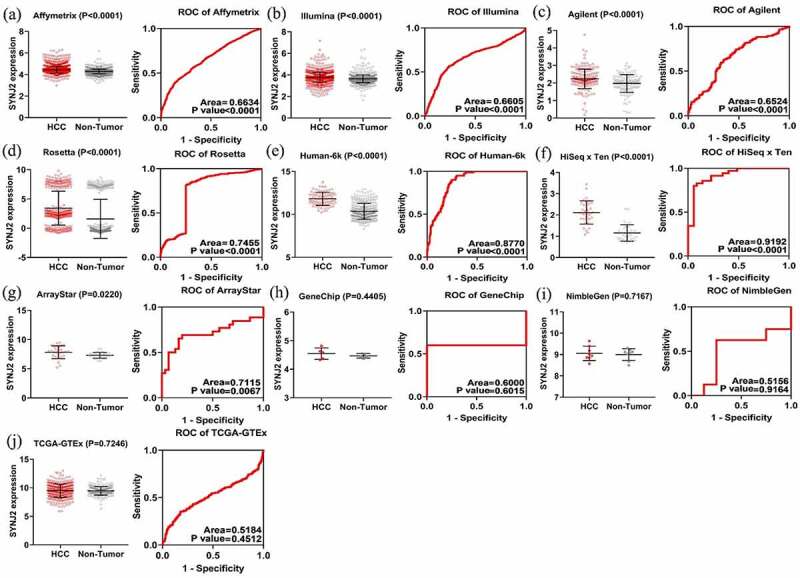
**Levels and ROC diagrams of merged SYNJ2 expression data**. Scatter plots and ROCs of merged SYNJ2 expression in (a) Affymetrix platform, (b) Illumina platform, (c) Agilent platform, (d) Rosetta platform, (e) Human-6k platform, (f) HiSeq×Ten platform, (g) ArrayStar platform, (h) GeneChip platform, (i) NimbleGen platform, and (j) TCGA-GTEx platforms

Following the aforementioned inclusion criteria, we ultimately obtained data from a total of 68 SYNJ2-related microarrays and high-throughput sequencing datasets from 11 detection platforms (Table 2). After correcting the merged expression data for batch effects, the levels and diagnostic accuracy of SYNJ2 in HCC were calculated, and the results are illustrated in Figure 4. Upregulated SYNJ2 in HCC was verified as significant for seven processes, and the AUCs of SYNJ2 ranged from 0.6524 to 0.9192.

**Table 2.** Characteristics of the merged datasets

	Dataset	HCC			Non-HCC		
ID	N	N	Mean	SD	N	Mean	SD
Affymetrix	28	1598	4.4432	0.3196	1316	4.2891	0.2163
Illumina	24	1061	3.7715	0.4654	770	3.6347	0.3562
Agilent	7	176	2.2357	0.5557	121	1.9751	0.5047
Rossetta	2	368	3.4306	2.9036	386	1.5999	3.3328
Human-6k	1	80	11.8181	0.7699	307	10.3700	0.9203
HiSeq × Ten	1	35	2.1138	0.5467	35	1.1540	0.3877
ArrayStar	1	26	7.8364	1.0932	30	7.3109	0.5077
GeneChip	1	5	4.5455	0.1995	5	4.4678	0.0776
NimbleGen	1	8	9.0550	0.3358	8	8.9982	0.2747
TCGA-GTEx	2	371	9.5031	1.1708	225	9.4723	0.7510

HCC: hepatocellular carcinoma. SD: standard deviation.

## Analysis of combined expression data

**Figure 5.**
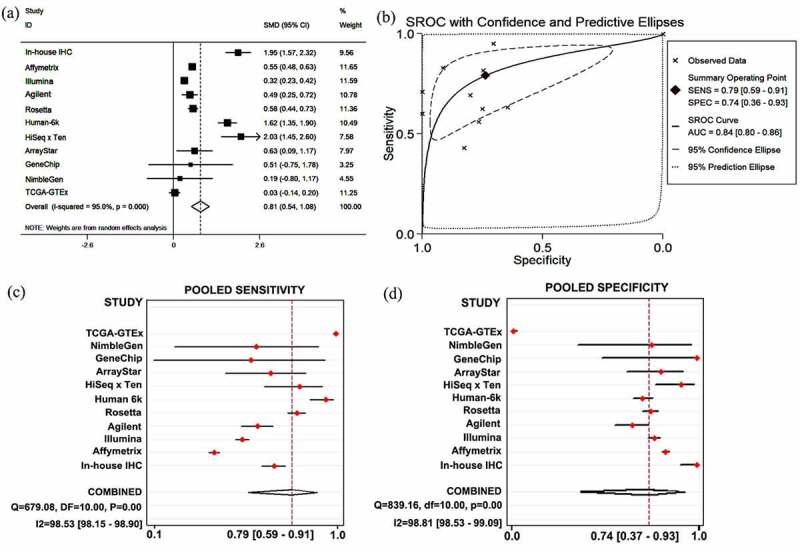
**Results of SYNJ2 levels and diagnostic value**. (a) Forest plots for SYNJ2 levels in HCC based on in-house IHC and different platforms. (b) SROC curve demonstrating performance of SYNJ2 in diagnosing HCC. (c) Pooled sensitivity and (d) specificity forest plots of SYNJ2

We performed a systematic review and data extraction to achieve more comprehensive results of SYNJ2 levels in HCC. In-house IHC data (285 HCC and 37 non-HCC tissues) and public expression data (3,728 HCC and 3,203 non-HCC tissues) were screened. Finally, significantly increased SYNJ2 in HCC tissues compared with noncancerous tissues was confirmed based on a forest plot with an SMD of 0.81 (95% CI: 0.54–1.08), and the area under the SROC was 0.84 (95% CI: 0.80–0.86). The pooled sensitivity and specificity of the included studies were 0.79 (95% CI: 0.59–0.91) and 0.74 (95% CI: 0.37–0.93), respectively (Figure 5).

## Genetic alterations in SYNJ2 in HCC

**Figure 6.**
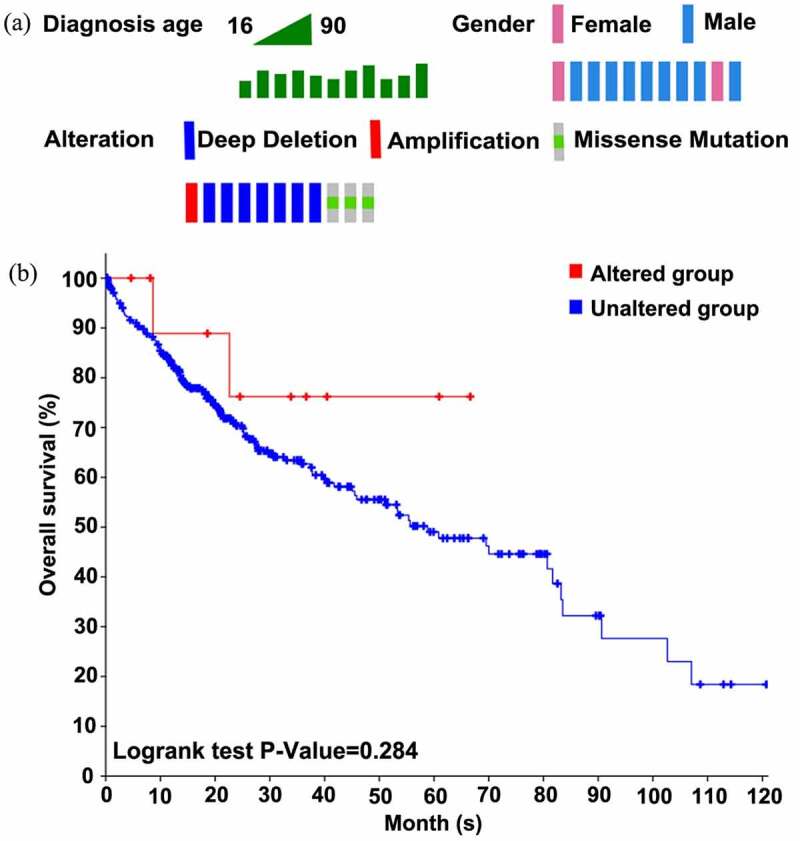
**Alterations and overall survival status of SYNJ2 in HCC**. (a) Diagnostic age, gender, and SYNJ2 altered types of HCC patients. (b) Difference of overall survival rates between SYNJ2 altered and unaltered groups

The genetic alterations of SYNJ2 were analyzed using the cBioportal database. We found that SYNJ2 was altered in 3% of patients (11/366), including nine male and two female patients. Missense mutations (three cases), amplification (one case), and deep deletion (seven cases) were discovered as three types of SYNJ2 alterations in HCC. The age of patients with altered SYNJ2 ranged from 49 to 80 years (Figure 6(a)). Moreover, the patients in the SYNJ2-unaltered group showed better overall survival outcomes than those in the SYNJ2-altered group (Figure 6(b)). In addition to dysregulated levels, mutated SYNJ2 may also worsen the prognosis of HCC cases and should be considered as a latent risk factor.

## Enrichment analysis of the differentially coexpressed genes of SYNJ2 in HCC

**Figure 7.**
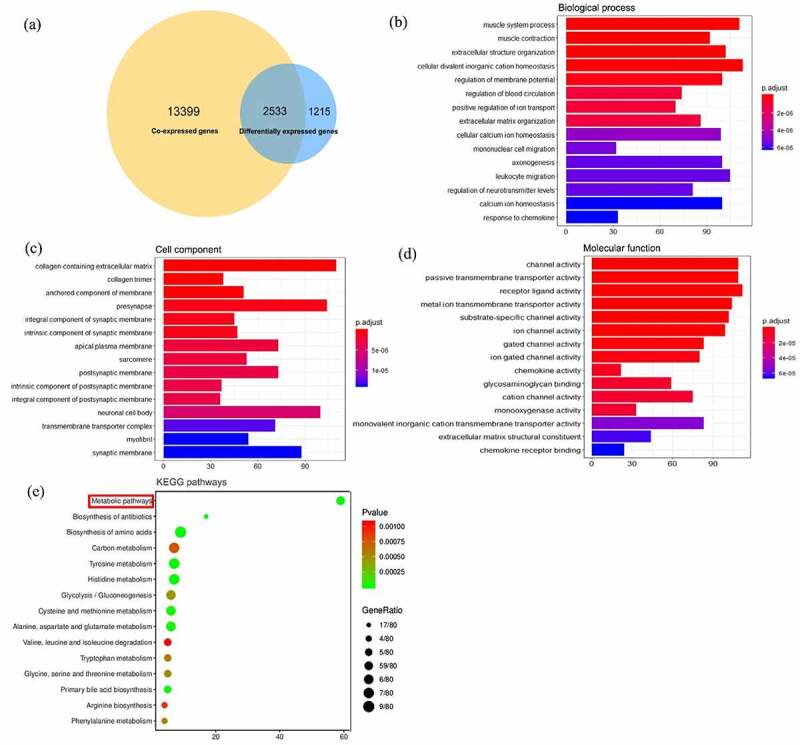
**Enrichment analysis of differentially coexpressed genes of SYNJ2**. (a) Venn diagram of coexpressed genes and differentially expressed genes. (b) Bar plots of biological process, (c) cell component, and (d) molecular function. (e) KEGG pathways of co-functioned SYNJ2 and differentially coexpressed genes. X axis represents the counts of involved genes and Y axis means enriched annotations and pathways

We separately obtained 3,744 differentially expressed genes (1,477 upregulated and 2,267 downregulated genes) and 15,928 coexpressed genes of SYNJ2 (|r| ≥ 0.8) from the extracted datasets. A total of 2,533 genes simultaneously appearing as both differentially expressed and coexpressed genes were identified as differentially coexpressed genes (DCGs) (Figure 7(a)). Gene ontology (GO) analysis diagrams of SYNJ2-related DCGs were constructed, and the top 15 annotations are shown, including the biological process (BP) (Figure 7(b)), cell component (CC) (Figure 7(c)), and molecular function (MF) terms (Figure 7(d)). The SYNJ2-related DCGs were found to function in many vital channel activities, such as receptor–ligand activity, ion gated channel activity, and chemokine activity. In the BP term analysis, SYNJ2 along with the DCGs were found to be involved in three significant processes, including organic hydroxy compound catabolic processes (P = 0.0083), small molecule catabolic processes (P = 0.0107), and alcohol catabolic processes (P = 0.0433). We considered 80 genes involved in these processes to uncover the potential signaling pathways and displayed the 10 most significant terms (Table 3). Surprisingly, together with the other 58 DCGs, the most significant enriched pathway that SYNJ2 was involved in was metabolism-related pathways (P < 0.0001) (Figure 7(e)).

**Table 3.** Top 10 significant Kyoto Encyclopedia of Genes and Genome pathways of SYNJ2 and differentially coexpressed genes

Term	Count	Genes	Fold Enrichment	P-value	FDR
Metabolic pathways	59	HEXA, HEXB, IL4I1, GLDC, ASPA, TDO2, SCLY, MAT1A, CYP7A1, HDC, GSTZ1, **SYNJ2**, HPD, AADAT, HMGCLL1, CYP1A1, ACADS, HAL, ALDH3B2, CYP26A1, ACADL, TAT, GLUL, CTH, SDS, AKR1B10, HAO2, UROC1, AKR1D1, OAT, PRODH, BCAT1, FUT5, AASS, KMO, GLS2, ARG1, MTHFS, CSAD, ADH4, HK3, ALDH4A1, ENO3, CDA, FUT2, INPP5B, BCKDHA, NOS1, AMACR, FTCD, ACACB, DBH, PCK1, CEL, AMDHD1, GCK, AGXT2, CYP4F2, CBS	4.8963	6.60E-35	7.41E-32
Biosynthesis of antibiotics	17	AADAT, BCAT1, BCKDHA, AASS, TAT, PCK1, GLDC, ARG1, CTH, GCK, SDS, HK3, HAO2, ENO3, OAT, CBS, PRODH	8.1120	1.05E-10	1.19E-07
Histidine metabolism	7	AMDHD1, ASPA, HDC, FTCD, HAL, ALDH3B2, UROC1	32.1878	4.49E-08	5.04E-05
Biosynthesis of amino acids	9	BCAT1, ARG1, GLUL, CTH, MAT1A, SDS, ENO3, TAT, CBS	12.6452	3.86E-07	4.34E-04
Tyrosine metabolism	7	ADH4, GSTZ1, ALDH3B2, IL4I1, DBH, TAT, HPD	20.2324	8.84E-07	9.94E-04
Primary bile acid biosynthesis	5	CYP39A1, CYP46A1, CYP7A1, AMACR, AKR1D1	29.7535	1.78E-05	0.0200
Alanine, aspartate and glutamate metabolism	6	GLS2, GLUL, ASPA, ALDH4A1, IL4I1, AGXT2	17.3420	1.95E-05	0.0219
Cysteine and methionine metabolism	6	CTH, MAT1A, SDS, IL4I1, TAT, CBS	15.9729	2.95E-05	0.0332
Glycolysis/Gluconeogenesis	6	GCK, ADH4, HK3, ENO3, ALDH3B2, PCK1	9.0593	4.56E-04	0.5119
Glycine, serine and threonine metabolism	5	CTH, SDS, AGXT2, CBS, GLDC	12.9695	5.23E-04	0.5865

FDR: false discovery rate.

## Interactome study of SYNJ2-related DCGs

**Figure 8.**
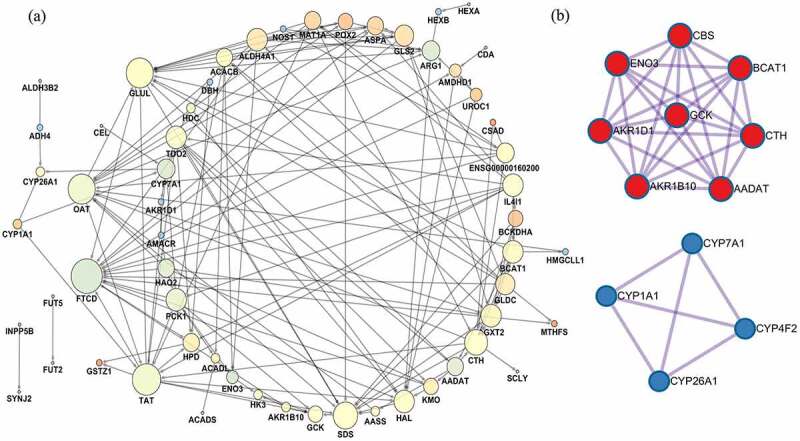
**Diverse interactions of proteins**. (a) Protein–protein interaction networks of metabolism-related differentially coexpressed genes. Each node means a gene and the bigger node size represents the higher interaction degree. The brighter node color represents the higher clustering coefficient. (b) Highly interconnected protein complexes

**Figure 9.**
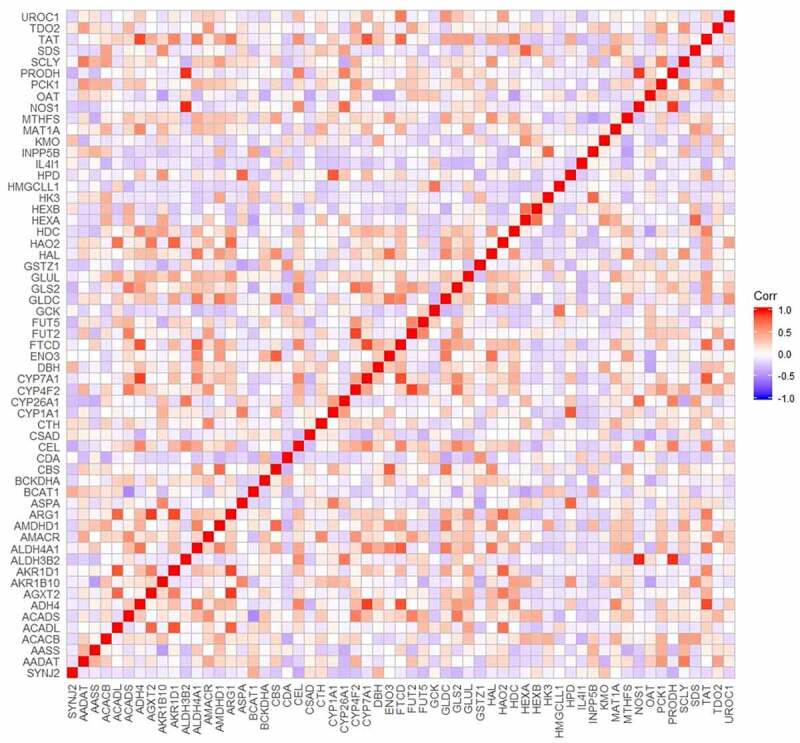
Correlations of metabolism pathways related differentially coexpressed genes based on CCLE

PPI analyses of the genes involved in the metabolic pathways were performed. As shown in Figure 8(a), a total of 151 connections were found among 59 DCGs, and the PPI enrichment P-value was less than 0.0001. The combined score of SYNJ2 and inositol polyphosphate-5-phosphatase B (INPP5B) was calculated as 0.837. Based on the MCODE clustering algorithm, both densely interconnected protein complexes and protein families constructed by DCGs in metabolic pathways were mapped (Figure 8(b)). Meanwhile, the correlations of 59 DCGs in 25 liver malignancy-related cell lines were estimated and visualized based on the CCLE database (Figure 9). Expression of SYNJ2 and INPP5B also showed a positive correlation among various liver malignant cells.

## TF-target regulations of SYNJ2 and DCGs in metabolic pathways

SYNJ2 and INPP5B were simultaneously found to interact in metabolic pathways. We investigated the TFs of SYNJ2 and INPP5B in the liver and showed the TFs that were validated in more than two ChIP-seq datasets (Table 4). Interestingly, four overlapped TFs appeared, including CCCTC-binding factor (CTCF), cAMP responsive element binding protein 1 (CREB1), GA binding protein transcription factor subunit alpha (GABPA), and RNA polymerase II subunit A (POLR2A). For a deeper understanding of the precise regulatory relationships of the TFs targets in certain pathways or diseases, genetics-related disease databases were explored, and based on the genetic association database (GAD, http://geneticassociationdb.nih.gov) we found that both CTCF and POLR2A could directly induce transcriptomic changes of metabolic pathways (Table 5). Thus, as the TFs of SYNJ2 and INPP5B, we speculated that CTCF and POLR2A could influence the metabolic pathways via modulating transcription processes and activities.

**Table 4.** Validated TF-target relationships of SYNJ2 and INPP5B in liver

Target	No. of datasets	Transcription factor	The peak close to transcription starting site			
			Chromesome	Peak start	Peak end	Enrichment score for the peak
SYNJ2	6	CTCF	Chr19	45,079,122	45,079,256	4.63
	4	CREB1	Chr19	45,079,135	45,079,311	30
	4	GABPA	Chr19	45,079,129	45,079,489	141
	3	POLR2A	Chr19	45,079,053	45,079,364	9.69
INPP5B	8	CTCF	Chr1	37,940,513	37,940,620	5.23
	5	CREB1	Chr1	37,946,977	37,947,186	6.11
	4	GABPA	Chr1	37,947,007	37,947,156	3.74
	4	POLR2A	Chr1	37,946,764	37,947,036	5.09
	3	HNF4A	Chr1	37,947,028	37,947,145	4.23

TF: transcription factor.

**Table 5.** Genetic diseases related to transcription factors of SYNJ2 and INPP5B

	Database				
Transcription factor	GAD	FunDO	NHGRI GWAS Catalog	KEGG DISEASE	OMIM
CTCF	Metabolic, HDL cholesterol, HDL lipoproteins	Lung cancer, Nephroblastoma, Breast cancer	HDL cholesterol	Mental and behavioral disorders, Autosomal dominant mental retardation	Mental retardation, autosomal dominant 21
CREB1	Pharmacogenomic, Suicide	-	-	-	Histiocytoma, angiomatoid fibrous, somatic
POLR2A	Metabolic, Triglycerides, Sex hormone-binding globulin	Adenovirus infection	-	-	-
GABPA	-	-	-	-	-

## SYNJ2 protein levels in HCC

**Figure 10.**
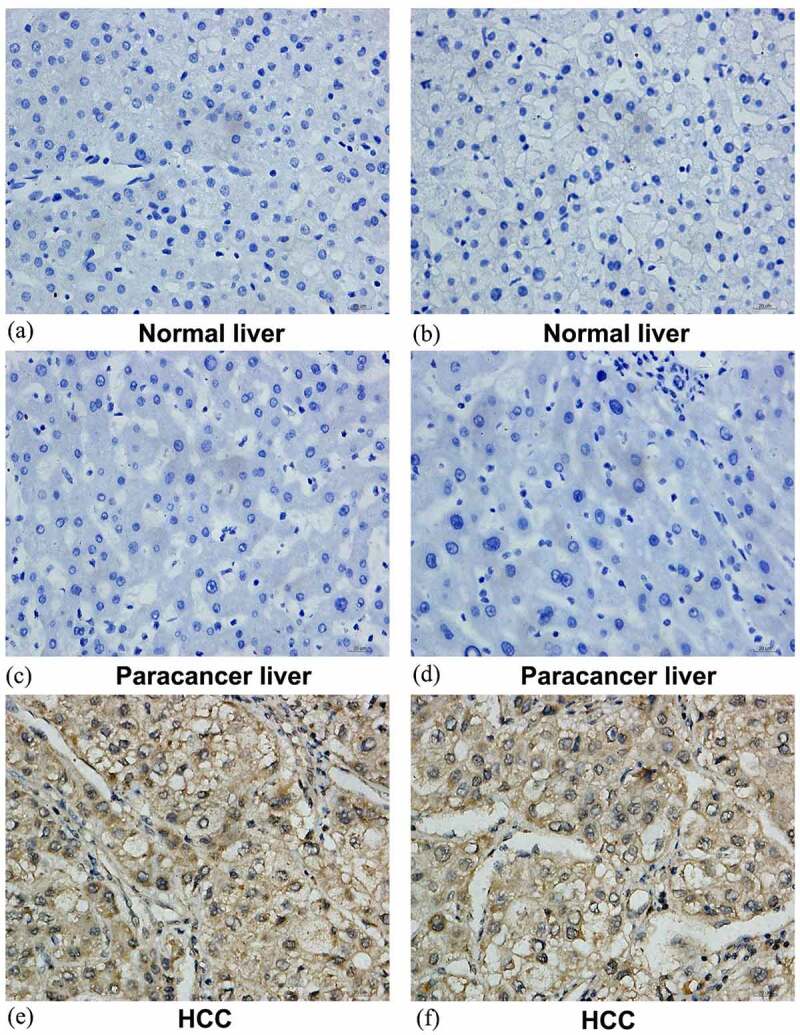
**Representative images of SYNJ2 protein expression**. Negative SYNJ2 IHC staining in normal liver (a, b) and tumor adjacent liver tissues (c, d). Positive diffused staining of SYNJ2 in HCC cells (e, f). The original images were magnified 400 times

**Figure 11.**
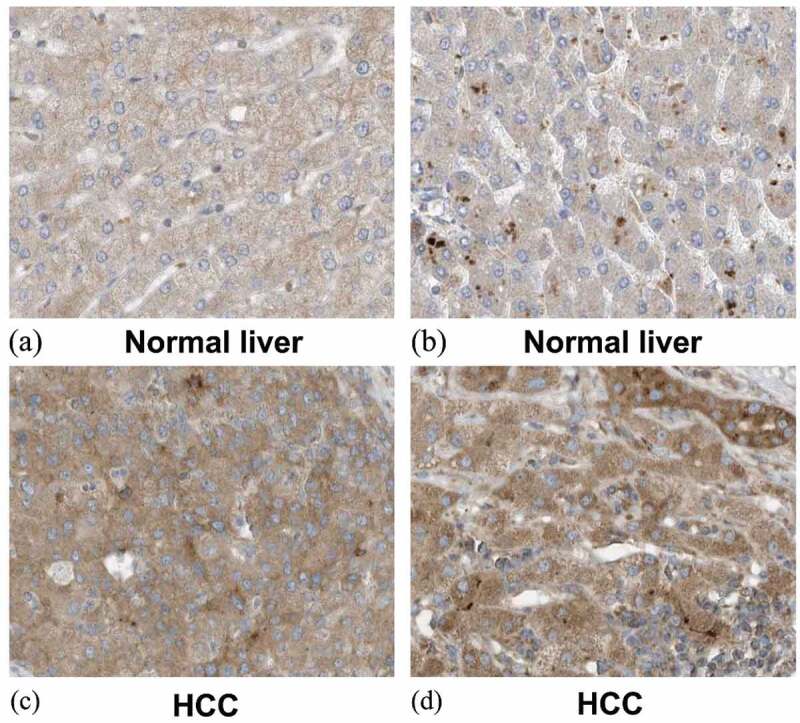
**Expression of SYNJ2 in normal liver and HCC from the Human Protein Atlas database**. (a, b) Low diffused staining of SYNJ2 in cytoplasmic and membranous of hepatocytes from samples 3402 and 2429. (c, d) Diffusedly brown medium staining of SYNJ2 in HCC tissues from patient 3196. The original images were magnified 400 times

The protein levels of SYNJ2 in HCC were investigated using in-house IHC and the human protein atlas (HPA) public database. The IHC staining of SYNJ2 in normal liver (Figure 10(a, b)), noncancerous liver (Figure 10(c, d)), and HCC (Figure 10(e, f)) tissues is shown (magnification, 400X). The protein levels of SYNJ2 in HCC tissues were greatly increased compared with those in noncancerous tissues. Additionally, the IHC staining patterns from the HPA database revealed higher SYNJ2 protein expression in HCC than in normal liver tissues. Weak-intensity and moderate-intensity SYNJ2 staining were found in liver hepatocytes (Figure 11(a, b)) and HCC (Figure 11(c, d)) (magnification, 400X), respectively. The protein level of increased SYNJ2 was validated to be consistent with the mRNA level in HCC tissues.

## Discussion

HCC is one of the most malignant tumors and poses a direct threat to human health and life. Nevertheless, developing novel drugs for HCC treatment remains difficult [20]. The mechanisms of HCC occurrence and progression are complex, including genetic alterations, diverse abnormalities of signaling pathways, and inflammatory responses [21]. In recent years, molecular targeted therapy has come to offer a new perspective for HCC treatment. Thus, it is extremely urgent to focus on the investigation of potential key targets in HCC.

This was the first study to mine SYNJ2 mRNA expression and the protein level data and perform experimental validation. As an alternatively spliced isoform of SYNJ1, SYNJ2 is widely expressed in various tissues. The levels of SYNJ2 were significantly increased in HCC patients, and SYNJ2 could function as a predictive marker with excellent discriminatory ability. As a member of the inositol-polyphosphate 5-phosphatase family, the encoded SYNJ2 protein inhibits clathrin-mediated endocytosis (CME) via translocation to the plasma membrane by interacting with ras-related C3 botulinum toxin substrate 1. CME is considered a crucial process modulating cell surface signaling and intercellular environment changes via the formation of endocytic vesicles, which are responsible for the uptaking and ingestion of numerous molecules from the cell surface to inside the cell [22,23]. Several classic clathrin-dependent endocytic pathways have been reported, such as the phosphatidylinositol-4,5-bisphosphate pathway, which was found to function jointly with the clathrin adaptor protein epsins in coated pits and accelerate vesicle formation. The clathrin-binding adaptor AP2 was revealed to directly bind to clathrin and cargo proteins to initiate the CME process [24]. As a novel Rac family small GTPase 1 (RAC1) effector molecule, SYNJ2 may also regulate RAC1-mediated endocytosis inhibitory effects and arrest cell growth [9]. Moreover, the hydrolysis reaction that occurs between the substrate and the 5′-phosphatase domain of SYNJ2 was demonstrated to be essential for clathrin-coated pit formation in the early phase, and the deletion of SYNJ2 inhibited receptor internalization of cells [10]. To summarize, all these findings indicate that the dysregulated CME process would interfere with targeted delivery and cell growth. We speculate that upregulated SYNJ2 can excessively function on certain signal transduction pathways of the CME process, which are closely related to the initiation and development of HCC. The details of the interacting proteins and potentially related mechanisms of SYNJ2 and CME in HCC still need to be investigated.

Genetic mutations are considered to intimately affect the morbidity and mortality of cancers by modulating genetic heterogeneity, signaling pathways, cellular processes, and so on [25]. In HCC, SYNJ2 has mainly been identified in cases of missense mutations, amplifications, and deep deletions. In terms of overall survival, patients with SYNJ2 alterations have poorer outcomes than those with wild-type SYNJ2. To date, several types of SYNJ2 mutations have been found to be crucial for functioning in different malignancies. In anaplastic intracranial ependymomas, deletion within the SYNJ2 loci may inhibit tumor invasion and may be a marker of good prognosis [26]. Moreover, homozygous deletion of SYNJ2 was detected in prostate cancer cells, and increases in SYNJ2 copies induced decreased breast cancer patient survival [18,27]. In HCC, mutation types of SYNJ2 should be detected in more clinical samples and there is much to be learned about the biological changes caused by different SYNJ2 mutation types. Prognosis might be improved via certain mutations of SYNJ2 in HCC.

From the results of the GO analysis, SYNJ2-related DCGs were related to three significant BP terms, including organic hydroxy compound catabolic processes, small molecule catabolic processes, and alcohol catabolic processes. Previous studies have revealed that 3-hydroxy-2-butanone can be utilized as a marker in exhaled breath for the early detection of HCC [28]. Furthermore, alcohol and diverse small molecules are reported to be closely correlated with the occurrence and treatment of HCC [29–32]. The enriched cellular component terms indicated that the DCGs could modulate components of the postsynaptic and synaptic membranes, which implies that the DCGs may also be involved in intercellular signaling and cargo protein interactions during the CME process. We also found many DCGs that play important roles in signal transduction, molecule transport across extracellular/intracellular membranes via proteins, and metal ion transmembrane transport, such as annexin A2 (ANXA2), the ATP binding cassette subfamily C member 8/9 (ABCC8/9), and potassium or calcium voltage-gated channel-related genes (KCNB1 and CACNA1H). Many molecular functions of DCGs were found to be highly associated with energy transformation and catabolism. Previous studies have reported that energy imbalance could be considered as risk factor of prognosis and medical treatment strategies for cancers, including triple-negative breast cancer, breast cancer, liver cancer, and colon cancer [33–37]. SYNJ2 has been verified to specifically and directly bind to the small GTPase Rac1 and may contribute to Rho GTPase regulation of cell proliferation and adhesion [9,11,38]. However, aberrant levels of SYNJ2 and the cofunctions of DCGs in HCC may break the interactions between SYNJ2 and Rac1 and create an unstable energy environment that can induce rapid HCC cell growth. Taken together, the underlying cofunctions of DCGs and SYNJ2 may potentially facilitate the progression of HCC via imbalance energy-related pathways.

Based on the KEGG signaling pathway analysis, SYNJ2, along with 58 other DCGs, was confirmed to be engaged in metabolic pathways. As a hallmark of cancer, dysregulated metabolic processes, such as reprogrammed energy metabolism, are widespread in cancer cells and can induce sustained cancer cell growth and division [39]. In the HCC microenvironment, multiple metabolic abnormalities coexist. Immune cells can be regulated directly or indirectly by metabolic interaction networks, and these complicated modulations maintain the stability of the immune microenvironment and affect the development of HCC [40]. In recent years, researchers have found that dysregulated fatty acid metabolism can induce oncogenic signaling pathway activation, which facilitates the initiation and progression of HCC [41]. In addition, specific metabolic targets need to be identified for use in novel HCC treatments, such as tricarboxylic acid cycle enzymes and key enzymes in related metabolic pathways [42–44]. At present, studies have found that cell proliferation, apoptosis, membrane homeostasis, and drug resistance are all related to lipid metabolism [45,46]. Cytoskeletal organization and F-actin polymerization, which participate in cell division, endocytosis, and cell migration, might be regulated by inositol lipids [47]. As a member of the inositol-polyphosphate 5-phosphatase family, SYNJ2 may play an important role in modulating cell growth and chemotherapy response in HCC.

In protein interaction networks, inositol polyphosphate-5-phosphatase B (INPP5B) specifically binds to SYNJ2. INPP5B and SYNJ2 are from the same gene family and could potentially control the motility of glioblastoma cells [46]. As one of the verified TFs of both SYNJ2 and INPP5B, CTCF was observed as a key modulator of hepatocytes repopulation and metabolism regulation via increased binding at promoters [48]. We supposed that dysregulated metabolic programs are closely associated with the CTCF-SYNJ2/INPP5B transcriptional regulatory axis in HCC. Moreover, promoter POLR2A occupancy and epigenetic states would influence cell-specific gene expression levels [49]. Aberrantly expressed levels of SYNJ2 in HCC might be induced by promoter binding of POLR2A. Transcription factors play crucial roles in the process of converting or transcribing DNA into RNA. They allow for unique expression of targeted genes in different types of cells and during development. Thus, CTCF and POLR2A may mediate the expression of targeted SYNJ2 and INPP5B. Once SYNJ2 levels dysregulate, the transcription regulatory axis would fail to regulate the metabolic process and induce HCC.

Based on the MCODE model, we found a cluster of cytochrome P450 family (CYP) members that can metabolize fatty acids and carcinogens, as well as participate in drug metabolism. Many studies have verified that CYP members, including CYP4F2, CYP4A11, and CYP2E1, can be potential prognostic factors in HCC [50–52]. In another cluster, aldo-keto reductase family 1 member D1 (AKR1D1) and aldo-keto reductase family 1 member B10 (AKR1B10), which are related to hepatic dysfunction and liver carcinogenesis, were discovered to interact with other genes. This cluster may trigger HCC based on the failure of glucose uptake and conversion to glycogen via suppression of glucokinase (GCK) [53]. We hypothesized that cofunctions among DCGs would be indirectly interfered with by upregulated SYNJ2 and accelerate the development of HCC. To develop a full picture of the interactions among DCGs, additional studies will be needed.

## Conclusions

The present study was first designed to investigate the full-scale mRNA and protein levels of SYNJ2 in HCC. Up-regulated SYNJ2 was identified via both public data repository and in-house experimental validation. The prognosis of SYNJ2-related HCC cases was successfully predicted using a nomogram model. Based on the SYNJ2-related differentially coexpressed genes, potential biological functions and signaling pathways were identified, which offered new insights into the role of SYNJ2 in metabolism. Furthermore, the critical underlying transcriptional axis of CTCF/POLR2A – SYNJ2/INPP5B (TF – target) in metabolic programs was discovered based on ChIP-seq datasets. In summary, we speculated that CTCF/POLR2A could directly dysregulate SYNJ2 levels and that increased SYNJ2 would affect HCC development via metabolic perturbation pathways. Upregulated SYNJ2 could also be used to predict the prognosis of HCC patients. The major limitation of this study is the absence of further validation of the detailed regulatory mechanisms of SYNJ2 and key DCGs. Notwithstanding this limitation, these valuable findings will be of interest to researchers studying potential pathogenic mechanisms of HCC and targeted treatments for HCC patients.

## Data Availability

The data used to support the findings during the current study are available from the corresponding author upon reasonable request.

## Research highlights

- First study to find increased levels of SYNJ2 in HCC tissues.
- SYNJ2 interacts with INPP5B in regulating the metabolic process in HCC.
- Underlying CTCF/POLR2A-SYNJ2/INPP5B axis plays a vital part in the metabolism of HCC.
